# Altered Functional Connectivity in Patients with Subcortical Vascular Cognitive Impairment—A Resting-State Functional Magnetic Resonance Imaging Study

**DOI:** 10.1371/journal.pone.0138180

**Published:** 2015-09-16

**Authors:** Weina Ding, Wenwei Cao, Yao Wang, Yawen Sun, Xue Chen, Yan Zhou, Qun Xu, Jianrong Xu

**Affiliations:** 1 Department of Radiology, Ren Ji Hospital, School of Medicine, Shanghai Jiao Tong University, Shanghai, 200127, P.R. China; 2 Department of Neurology, Ren Ji Hospital, School of Medicine, Shanghai Jiao Tong University, Shanghai, 200127, P.R. China; Institute of Psychology, Chinese Academy of Sciences, CHINA

## Abstract

Recent neuroimaging studies have shown that people with subcortical vascular cognitive impairment (sVCI) have structural and functional abnormalities in the frontal lobe and subcortical brain sites. In this study, we used seed-based resting-state functional connectivity (rsFC) analysis and voxel-mirrored homotopic connectivity (VMHC) techniques to investigate the alteration of rsFC in patients with sVCI. rsFC and structural magnetic resonance images were acquired for 51 patients with subcortical cerebrovascular disease. All patients were subdivided based on cognitive status into 29 with sVCI and 22 controls; patient characteristics were matched. rsFC of the posterior cingulate cortex (PCC) and VMHC were calculated separately, and rsFC of the PCC and VMHC between the two groups were compared. The regions showing abnormal rsFC of the PCC or VMHC in sVCI patients were adopted as regions of interest for correlation analyses. Our results are as follows: The patients with sVCI exhibited increases in rsFC in the left middle temporal lobe, right inferior temporal lobe and left superior frontal gyrus, and significant decreases in rsFC of the left thalamus with the PCC. sVCI patients showed a significant deficit in VMHC between the bilateral lingual gyrus, putamen, and precentral gyrus. Additionally, the z-memory score was significantly positively associated with connectivity between the left thalamus and the PCC (r = 0.41, p = 0.03, uncorrected) in the sVCI group. Our findings suggest that the frontal lobe and subcortical brain sites play an important role in the pathogenesis of sVCI. Furthermore, rsFC between the left thalamus and the PCC might indicate the severity of sVCI.

## Introduction

The dramatic worldwide increase in the proportion of elderly people has brought attention to aging-related cognitive impairments. Dementia is prominent among chronic diseases affecting elderly populations [1], and has emerged as a major global health problem [2, 3]. The overall prevalence of dementia in developed countries ranges from 5 to 10% in people >65 years old, and the prevalence of vascular dementia doubles every 5.3 years [4]. Vascular cognitive impairment (VCI) includes all levels of cognitive loss, from mild deficits in one or more cognitive domains to broad dementia syndrome caused by cerebral vessel disease [5]. The current classification of VCI subtypes includes vascular cognitive impairment with no dementia (VCIND), vascular dementia, and mixed primary neurodegenerative dementia (usually Alzheimer’s disease [AD]) with vascular dementia [6].

As one of the most common subtypes of VCI, subcortical VCI (sVCI) is caused by subcortical cerebrovascular disease, which appears as lacunar infarcts or extensive and/or diffuse lesions in the white matter on magnetic resonance imaging (MRI) [7]. Affected individuals typically show impaired executive functions with relative preservation of memory [8]. Dysexecutive syndrome of sVCI is characterized by symptoms, including decreased mental processing speed, decreased working memory, and impaired abstract reasoning, that are associated with lacunes and deep white-matter changes [9].

Many studies have revealed structural and functional abnormalities in VCI, espically sVCI patients, especially in the frontal–subcortical neuronal circuits [9–14]. Thong et al. [9] found cortical thinning in the frontal lobes of patients in the mild-to-severe sVCI groups, and the severity of this thinning was greater and more widespread than in patients without sVCI. Duering et al., [15] set out to identify a strategic brain network for processing speed by applying graph-based data-mining techniques to MRI lesion maps from patients with sVCI The network included lacunar lesions in the left anterior thalamic radiation and the left cingulum as well as white matter hyperintensities in the left forceps minor, the left parahippocampal white matter and the left corticospinal tract. Structural equation modeling confirmed the findings obtained from the Bayesian models. In summary, using graph-based models, they identified the strategic brain network with the highest predictive value for processing speed in a cohort of patients with cerebral autosomal dominant arteriopathy with subcortical infarcts and leukoencephalopathy. These findings confirm and extend previous results indicating the role of frontal–subcortical neuronal circuits (particularly, dorsolateral prefrontal circuits and the cingulum) in sVCI. Yi et al. [11] found widespread gray-matter atrophy in sVCI patients, which occurred primarily in the frontal brain regions and several subcortical brain sites.

Recently, resting-state functional (rsf) MRI has attracted much attention, and has been widely used to investigate the pathogenesis of neurological and psychiatric diseases. Likewise, functional connectivity (FC) methods based on low-frequency (0.01–0.08 Hz) spontaneous blood oxygenation level-dependent fluctuations in rsfMRI provide a powerful tool to characterize intrinsically functional associations among brain regions [16–18]. This technique enables the clinician to gain additional insight into the functional organization of the brain in patients with sVCI.

Functional homotopy, the high degree of correlated activity between homotopic interhemispheric counterparts, is one of the most salient aspects of the brain’s intrinsic functional architecture [19]. rsfMRI approaches, which reveal patterns of coherent spontaneous fluctuations in the fMRI signal, offer a means by which to directly quantify interhemispheric functional interactions. Interhemispheric connectivity reflects the process of exchange and the integration of information between the cerebral hemispheres [20]. A recently validated approach, referred to as “voxel-mirrored homotopic connectivity” (VMHC) [21], was introduced to quantify rsFC between each voxel in one hemisphere and its mirrored voxel in the opposite hemisphere. This method has been performed on patients with addiction, schizophrenia, chronic tinnitus, and depression [21–26].

rsfMRI is a technique that permits assessment of inter- and intrahemispheric FC. Our previous research revealed diffuse alteration of rsFC within the posterior cingulate cortex (PCC) in patients with VCIND [27], The present study uses rsfMRI to assess the integrity of interhemispheric interaction in these participants to determine whether this technique can provide additional evidence of the neuropathological mechanism of VCI; therefore, we mainly focused on confirming whether rsFC with PCC and VMHC were altered in patients with sVCI. Given evidence for structural and functional changes in the frontal lobe and subcortical brain sites (thalamus and caudate) associated with sVCI [9, 15], we expected these regions to be particularly affected. We also investigated whether rsFC and VMHC were related to clinical scores in sVCI patients.

## Materials and Methods

### Participants

The current study was approved by the Research Ethics Committee of Ren Ji Hospital, School of Medicine, Shanghai Jiao Tong University (China). Each patient gave his or her informed written consent. Seventy-five patients with subcortical cerebrovascular disease were recruited from the Neurology Department of Ren Ji Hospital from July 2012 to May 2014.

To be included in the study, patients with sVCI had to meet the following criteria [28]: (1) the patient or his/her caregiver made a subjective cognitive complaint; and (2) the patient had a subcortical vascular feature, including a focal neurological symptom, any suggestive sign of cerebrovascular disease, or significant white matter hyperintensities (WMH) or lacunar infarcts (as shown on MRI scans). The exclusion criteria were: cerebral hemorrhages; cortical and/or cortico–subcortical, non-lacunar territorial infarcts; watershed infarcts; specific causes of white-matter lesions (e.g., multiple sclerosis, sarcoidosis, and brain irradiation); neurodegenerative diseases (including AD and Parkinson’s disease); and signs of normal pressure hydrocephalus or alcoholic encephalopathy. Patients with a low level of education (<6 years), severe depression (Hamilton Depression Rating Scale [HDRS] ≥18) [29], severe claustrophobia, contraindications to MRI, other psychiatric comorbidities, or severe VCI (inability to perform neuropsychological tests) were also excluded. Patients with severe brain atrophy were excluded to avoid the effect of the atrophy on processing fMRI data. Finally, 51 of the recruited right-handed patients were subdivided based on cognitive status into either the subcortical cerebrovascular disease-normal cognitive group (control group, n = 22) or the sVCI group (n = 29) based on age, sex, and education level.

### Magnetic resonance imaging

MRI was conducted using the Signa HDxt 3T MRI scanner (GE Healthcare, USA). An eight-channel standard head coil with foam padding was used to restrict head motion. During rsfMRI, the patients were instructed to remain motionless, stay awake, keep their eyes closed, and avoid thinking of anything in particular. A gradient-echo echo-planar sequence was used. Thirty-four transverse slices (repetition time [TR] = 2000 ms, echo time [TE] = 30 ms, field of view [FOV] = 230×230 mm^2^, matrix = 64×64, slice thickness = 4 mm) aligned along the anterior commissure–posterior commissure line were acquired. Each rsfMRI scan lasted 440 s. Several other sequences were also acquired, including (1) 3D fast spoiled gradient recalled sequence images (TR = 6.1 ms, TE = 2.8 ms, TI = 450 ms, slice thickness = 1.0 mm, gap = 0, flip angle = 15°, FOV = 256×256 mm^2^, matrix = 256×256, slice thickness = 1 mm, number of slices = 166), (2) axial T1-weighted fast spin-echo sequences (TR = 331 ms, TE = 4.6 ms, FOV = 256×256 mm^2^, matrix = 512×512, slice thickness = 4mm, number of slices = 34), (3) axial T2-weighted fast spin-echo sequences (TR = 3013 ms, TE = 80 ms, FOV = 256×256 mm^2^, matrix = 512×512, slice thickness = 4 mm, number of slices = 34), and (4) T2-fluid attenuated inversion recovery sequences (TE = 150 ms, TR = 9075 ms, TI = 2250 ms, FOV = 256×256 mm^2^, matrix = 128×128, slice thickness = 2 mm, number of slices = 66).

### Magnetic resonance imaging analysis

Two visual rating scales for WMH severity were used on T2-weighted axial scans by two trained neurologists blinded to clinical data. Using the age-related white-matter changes (ARWMC) visual grading scale, the degree of WMH was rated on a four-point scale in five different regions of the right and left hemispheres (frontal, parieto-occipital, temporal, basal ganglia, and infratentorial) [30]. Basic ARWMC scores for each brain region were summed to obtain the total (t) ARWMC score, which was used as a measure of the entire brain WMH load.[30] The Fazekas visual rating scale was applied for separate assessment of periventricular and deep subcortical WMH in all patients [31].

### Neuropsychological assessment

Neuropsychological assessments were performed within 1 week of the MRI. None of the patients suffered a new clinical stroke or transient ischemic attack between the MRI and the assessment. The Montreal Cognitive Assessment (MoCA) [32] and Mini Mental State Examination (MMSE) [33], and a comprehensive battery of neuropsychological tests were designed based on a review of relevant published reports. Trail making test A and B, the Stroop color word test, verbal fluency (category) test, auditory verbal learning test (short-delayed and long-delayed free recall), Rey–Osterrieth complex figure test (delayed recall), Boston naming test (30 words), Rey–Osterrieth complex figure test (copy), Lawton and Brody’s activities of daily living (ADL) scale test, Barthel Index, HDRS, and neuropsychiatric inventory were included. To assess the cognitive status of patients, the scores for each measurement of normal elderly patients in Shanghai, China, were used as the normal baseline. Cognitive dysfunction was defined as −1.5 SD in at least one neuropsychological test.

#### Z-score calculation

To allow a direct comparison of different tests, z-scores were generated for the neuropsychological measures. A z-score defines where a score falls in the normal distribution of scores; a z-score of +1.0 corresponds to a score 1.0 SD above the mean score. Direct comparison of performance among tests was possible because z-scores for all tests were based on an identical control population. Raw scores for each of the neuropsychological tests were z-transformed. Thereafter, the z-score of each domain (memory, attention–executive, language, and visual space function) was generated by averaging z-scores of respective tests. Finally, composite Z-scores, indicating general intellect, were computed by averaging the z-scores of individual cognitive domains.

### Analysis of functional magnetic resonance imaging

Structural brain MRI scans (T1- and T2-weighted images) were inspected by two experienced neuroradiologists. All of the patients were found to have WMH and slight atrophy on the MRI; patients with severe brain atrophy were excluded.

As a central component of the proposed default mode network, the PCC is implicated in attentional processes [34]. A number of studies suggest that the PCC plays an essential role in spatial orientation, self-appraisal, and internal monitoring, as well as in memory processing [35]. Given that the PCC is the seed region most commonly used in studies of the default network, it was used as the region of interest (ROI) to study its altered connections with other brain regions. To calculate rsFC with the PCC, functional images were preprocessed using Data Processing Assistant for Resting-State Functional MR Imaging toolkit (DPARSF3.0 Advanced edition) [36], which synthesizes procedures in the Resting-State Functional MR imaging toolkit (REST; http://www.restfmri.net)[37], and SPM8 (www.fil.ion.ucl.ac.uk/spm). The first 10 images were excluded to ensure steady-state longitudinal magnetization; the remaining images were then corrected for temporal differences and head motion. After patient selection, neither translation nor rotation parameters in any given data set exceeded ±1.0 mm or ±1.0°. Moreover, the mean framewise displacement (FD) was computed by averaging FDi from every time point for each subject [38]. There were no differences in the mean FD between groups (p = 0.26). All normalized T1 images were averaged to generate a mean normalized T1 template. The rsFC calculation procedure was previously described [27], but our own T1 template was used in the present study. The PCC template was selected as the ROI because our previous research mentioned using WFU-Pick Atlas [27, 39]. The blood oxygenation level-dependent signal time series in the voxels within the seed region were averaged to generate the reference time series. For each subject and seed region, a correlation map was produced by computing the correlation coefficients between the reference time series and the time series from all other brain voxels. Correlation coefficients were then converted to z-values using the Fisher z-transformation to improve the normality of the distribution [40]. The individual z-scores were entered into SPM8 for a one-sample t-test to determine the brain regions with significant rsFC to PCC within each group.

The VMHC calculation procedure was previously described [25, 26]. Briefly, all normalized T1 images were averaged to generate a mean normalized T1 image, after which a nonlinear registration was used to normalize each brain to the symmetrical template. The transformation was applied to the symmetrical brain template to the normalized functional data. The VMHC value was then computed as the Pearson correlation (Fisher z-transformation) between the time series data of every pair of symmetrical voxels. Intergroup differences were compared using voxel wise t-tests.

### Statistical analyses

All demographics and neuropsychological data analyses were performed using SPSS version 19.0 (IBM Corporation, Armonk, NY, USA). Two-sample t-tests were performed for assessing group comparisons to determine the intergroup demographic differences, and an χ^2^ test was used for sex comparison. The Mann–Whitney U test was used for comparing continuous variables if data were not normally distributed. A two-tailed p-value of 0.05 was considered statistically significant for all analyses.

The statistical significance of rsFC and VMHC within groups was analyzed with a one-sample t test (p < 0.05, corrected with a single voxel height of p < 0.01 and cluster volume >594 mm^3^, p < 0.01 and cluster volume >486 mm^3^, respectively), using a software program (AFNI AlphaSim; http://afni.nimh.gov/pub/dist/doc/manual/AlphaSim.pdf).The significant differences in rsFC between groups were analyzed using a two-sample t test (p < 0.05, corrected with a single voxel height of p < 0.01 and a cluster volume >405 mm^3^) using AFNI AlphaSim and the significant differences in VMHC between groups were analyzed using a two-sample t test (p < 0.05, corrected with a single voxel height of p < 0.01 and a cluster volume > 432 mm^3^) using the same software program with a gray matter mask produced by the symmetric template previously outlined. Pearson correlations were performed to identify the relationship between the strength of connectivity with the clinical neuropsychological variables. To account for the potential influence of outliers, Shepherd’s pi correlation analysis was used [41]. Finally, a two-tailed p value of 0.007 with Bonferroni correction was considered statistically significant.

## Results

### Demographics, neuropsychological data, and magnetic resonance image analysis

The demographic characteristics, neuropsychological scores, and MRI analyses of the patients are presented in Table 1. Sex, age, and education level did not differ significantly between the two groups. The MoCA, MMSE, composite z-score, and z-scores of the each domain (memory, attention–executive, language, and visual space function) of the sVCI group were significantly lower than those of the controls. The tARWMC and Fazekas PV scores were significantly higher in the sVCI group.

**Table 1:** Demographics, neuropsychological data, and magnetic resonance imaging analysis in the control group and vascular cognitive impairment group.

	sVCI (n = 29)	CON (n = 22)	p value
Sex, male/female	19/10	14/8	0.41
Age (year)	71.07±6.72	67.78±6.75	0.09
Education(year)	10.40±3.42	10.74±3.03	0.72
MMSE	25.90±3.09	28.61±1.23	<0.001
MoCA	20.14±5.84	25.48±2.68	<0.001
composite *Z*-score	-1.39±1.39	0.29±0.55	<0.001
Memory z-score	-1.39±0.98	-0.17±0.54	<0.001
Attention–executive z-score	-1.76±1.72	1.72±0.56	<0.001
Language z-score	-1.17±1.89	0.14±1.45	0.01
Visual space z-score	-0.33±2.19	1.20±0.31	0.002
tARWMC	12 (7–22)	8 (1–18)	<0.001
Fazekas PV score	2.31 (1–3)	1.52(0–3)	0.004
Fazekas DS score	2.20 (1–3)	1.78(1–3)	0.052

sVCI = subcortical vascular cognitive impairment; CON = control group (patients with subcortical vascular disease with no cognitive impairment); MoCA = Montreal Cognitive Assessment, MMSE = Mini Mental State Examination; tARWMC = total age-related white matter changes, PV = periventricular; DS = deep subcortical. Note: All data were given as mean ± SD except that tARWMC and Fazekas score data are given as median (range, [min, max]). Two-sample t-tests were performed for assessing group comparisons and the Mann–Whitney U test was used for comparing continuous variables if data were not normally distributed. p value < 0.05 was considered to be statistically significant.

### Group differences in resting-state functional connectivity with posterior cingulate cortex

**Fig 1:**
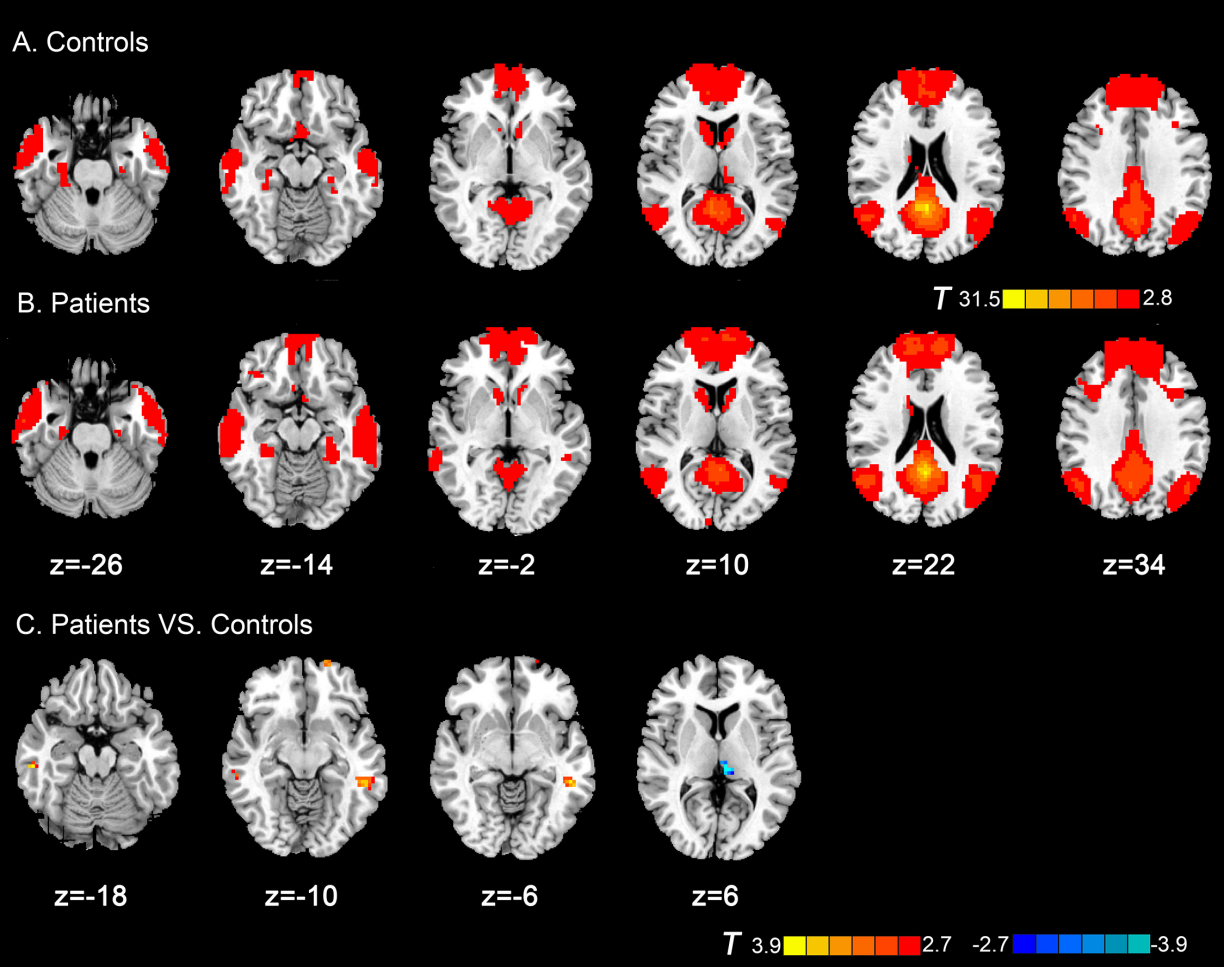
Axial magnetic resonance (MR) images show resting-state functional connectivity (rsFC) with posterior cingulate cortex within and between groups. Regions show significantly positive functional connectivity in (A) control subjects and (B) patients with subcortical sVCI (p < 0.05, AlphaSim corrected). (C) Compared with the control group, patients with sVCI exhibited increased rsFC in the left middle temporal lobe, right inferior temporal lobe and left superior frontal gyrus. The left thalamus exhibited decreased connectivity (p < 0.05, AlphaSim corrected). The t-score bars are shown on the right. Red indicates patients with sVCI > control and blue indicates patients with sVCI < control. Note: The left part of the figure represents the participant’s left side, the right part represents the participant’s right side. sVCI = subcortical vascular cognitive impairment.

Within-group of rsFC with PCC showed in Fig 1A and 1B. In the present study, we just focused on the positive correlations of PCC, that is, a traditional default mode network (DMN) study of the participants. As shown in Fig 1A and 1B the DMN consisted of the PCC, medial prefrontal cortex, left and right superior frontal gyrus, left and right lateral parietal cortex, left and right lateral temporal cortex, left and right parahippocampal gyrus, and thalamus, which is in agreement with the DMN identified in previous studies [42–44]. Then, we defined a PCC-FC mask from combing the results of within group *t* tests of sVCI and control groups to perform the between-group comparisons. Compared with the control group, patients with sVCI exhibited increased rsFC in the left middle temporal lobe, right inferior temporal lobe and left superior frontal gyrus. The left thalamus, exhibited decreased connectivity (Table 2 and Fig 1C).

**Table 2:** Regions showing group differences in FC with PCC.

Peak MNI coordinate region		Peak MNI coordinates			Number of cluster voxels	Peak *t* value
		x	y	z		
1	Left middle temporal lobe (BA21)	-54	-39	-6	56	3.68
2	Right inferior temporal lobe (BA20)	57	-27	-18	16	3.72
3	Left superior frontal gyrus (BA11)	-18	66	-12	15	3.61
4	Left thalamus	-9	-27	6	18	-4.16

MNI = Montreal Neurological Institute; sVCI = subcortical vascular cognitive impairment; BA = Brodmann area; Note: *t* >0 indicates sVCI group >control group in FC with PCC. (p < 0.05, AlphaSim- corrected)

### Group differences in voxel-mirrored homotopic connectivity

**Fig 2:**
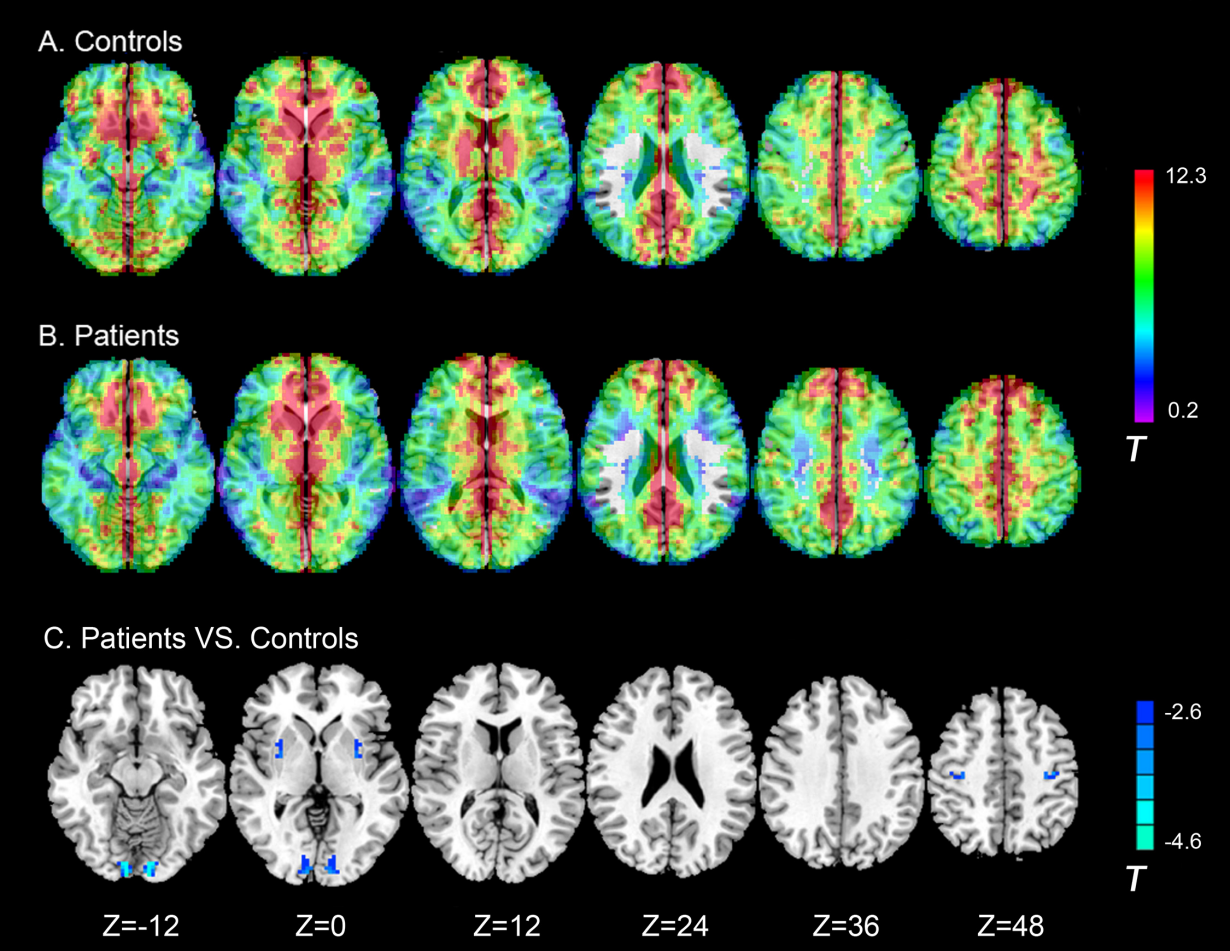
Axial MR images show interhemispheric functional connectivity within and between groups. Regions show significant interhemispheric functional connectivity in (A) patients with sVCI, and (B) control subjects (p < 0.05, AlphaSim corrected). C, Homotopic regions show decreased (Blue) functional connectivity in patient group (p < 0.05, AlphaSim corrected). Note: The left part of the figure represents the participant’s left side, the right part represents the participant’s right side. sVCI = subcortical vascular cognitive impairment.

Within-group results indicated that both patients (Fig 2A) and control subjects (Fig 2B) had robust homotopic functional connectivity with regional differences in strength. Although sVCI patients showed deficits in VMHC between the left and right lingual gyrus, putamen, and precentral gyrus. They did not show regional VMHC that was greater than that of the controls (Table 3 and Fig 2C).

**Table 3:** Regions showing group differences in voxel-mirrored homotopic connectivity.

Peak MNI coordinate region		Peak MNI coordinates			Number of cluster voxels	Peak *t* value
		x	y	z		
1	Lingual gyrus (BA18)	±9	-90	-9	73	-5.02
2	Putamen	±30	0	0	18	-3.44
3	Precentral gyrus (BA4)	±36	-18	51	25	-3.69

MNI = Montreal Neurological Institute; VMHC = voxel-mirrored homotopic connectivity; BA = Brodmann area; sVCI = subcortical vascular cognitive impairment; Note: *t* < 0 indicates sVCI group <control group in VMHC. (p < 0.05, AlphaSim-corrected).

### Relationship between resting-state functional connectivity of the posterior cingulate cortex and voxel-mirrored homotopic connectivity values with clinical variables

**Fig 3:**
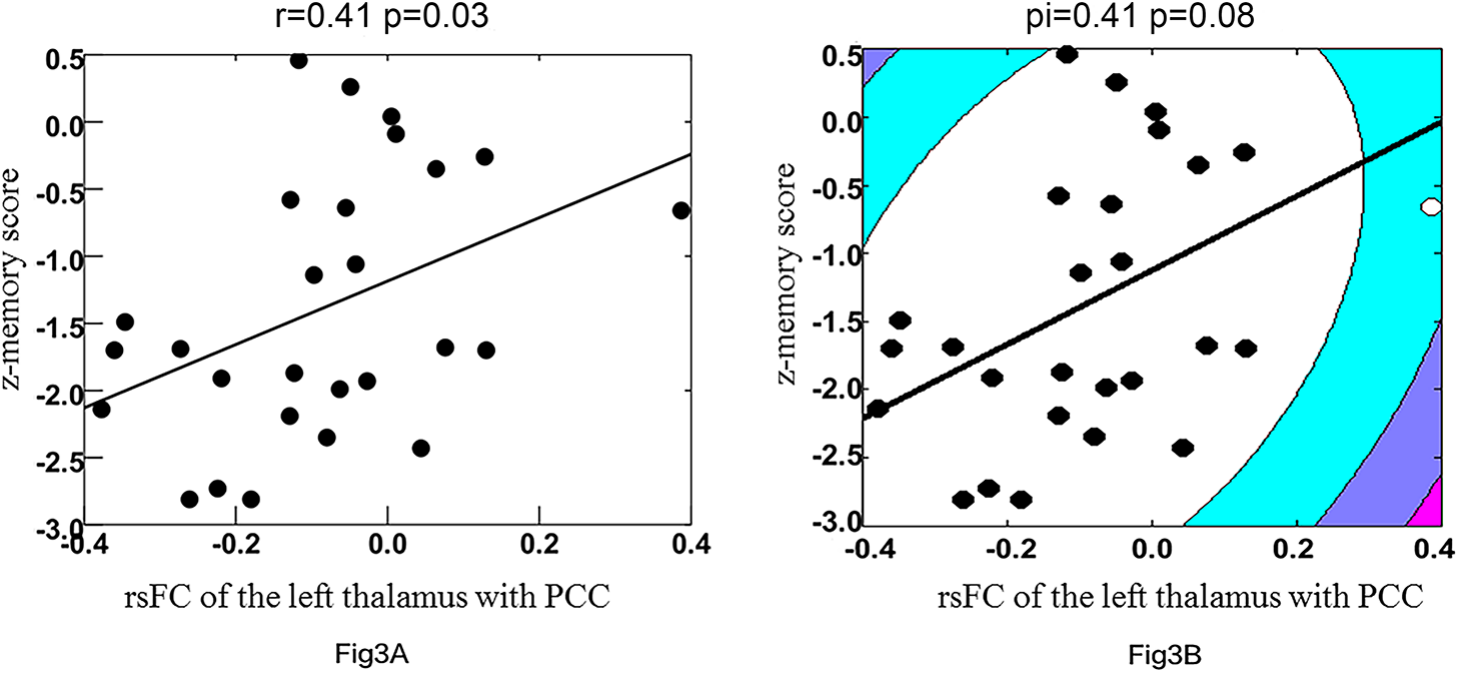
Correlation between resting-state functional connectivity (rsFC) between the left thalamus and posterior cingulate cortex and Z-memory score in the sVCI group. Outliers are indicated by open circles. 3A and 3B represent the best-fit line of Pearson’s and Shepherd’s correlations, respectively. sVCI = subcortical vascular cognitive impairment.

To identify the relationship between the strength of rsFC and VMHC and the clinical scores, the mean rsFC or VMHC values were extracted and averaged within a spherical 5.0-mm ROI radius centered on the rsFC or VMHC group difference peak (reported in Figs 1 and 2 and Tables 2 and 3). Pearson correlations were performed between rsFC or VMHC with MMSE, MoCA, composite z-score, and z-score of each domain within the sVCI group. A significantly positive correlation was observed only in rsFC between the left thalamus and PCC with z-memory score in the sVCI group (r = 0.41, p = 0.03) (Fig 3A), whereas no significant results were found using Shepherd’s correlation (Pi = 0.41, p = 0.07) (Fig 3B). No significant correlations survived after Bonferroni correction.

## Discussion

There are several causes of reduced interregional correlation, which, in turn, can be construed as dysfunctional connectivity. Some such causes are pathophysiological mechanisms operating within a specific region, such as abnormal synaptic modulation and signaling or loss in the amplitude of oscillations [45, 46]. Similarly, a loss in the homogeneity of oscillations of neuronal elements composing a regional node might also manifest as a reduction in interregional correlation [46, 47]. The mechanisms underlying the deficits in VMHC are still unknown, but they could be related to widespread white matter–integrity abnormalities [48], such as deficits in white-matter connectivity in the corpus callosum [49, 50], and dysfunctions in local gray-matter structure [22]. Additionally, alternative (e.g., subcortical) pathways are known to exist, although the callosum is the largest conduit for information transfer and coordination between the hemispheres. sVCI patients were found to have higher tARWMC and Fazekas scores than the control group. Sachdev et al. [51] reported that patients with post-stroke CI had a significantly higher load of total as well as periventricular WMH in terms of absolute volume. Sudo et al. [52] also found that mild VCI was significantly associated with the severity of the white-matter hyperintensities as graded on the Fazekas scale; the higher grades were more frequently associated with the development of cognitive impairments than the lower grades. Lin et al. [53] found that, compared with normally cognitive patients, VCIND decreased fractional anisotropy and increased mean diffusivity in all projection, association, and commissural fibers. Additionally, they found that MoCA scores correlated with diffusion tensor imaging values in all supratentorial WM tracts. It is presumed that more extensive and/or diffuse white-matter lesions have more of an effect on the rsFC and VMHC deficits, but the definite mechanisms need further study.

Different rsFC of the PCC were detected in patients with sVCI. Patients with sVCI exhibit increased rsFC in the left middle temporal lobe, right inferior temporal lobe and left superior frontal gyrus. The left thalamus exhibited decreased connectivity, which is partly consistent with our previous research [27]. In addition, deficits in VMHC were detected between left and right lingual gyrus, putamen, and precentral gyrus in sVCI patients. These changes might be caused by subcortical white-matter lesions that destroyed direct and indirect fiber tract connectivity across the cerebral white matter and influenced cortical rsFC and hypoperfusion resulting from subcortical cerebrovascular disease. This increased connectivity might be evoked by compensatory recruitment and plasticity mechanisms [27].

The lingual gyrus is a brain structure that is linked to vision processing, especially that related to letters. Ghosh [54] found that a cerebellar–occipital–thalamic network is activated when the semantic relatedness of words are assessed by the brain. Semantic association tasks, in addition to being a measure of language fluency, are an indication of the extent of executive function and working memory; therefore, these areas could play a role in the transfer of semantic information to a more permanent storage format in the cortical association areas. The thalamus is a crucial brain area that processes and integrates neural activity from widespread neocortical inputs and outputs [55] and is believed to coordinate information and facilitate communication (e.g., memory, attention, and perception) in a number of areas in the cerebral cortex [56, 57] The thalamus pulvinar has widespread connectivity with the posterior parietal lobe and the precuneus. Our results revealed a significantly positive correlation in rsFC of the left thalamus with a z-memory score in the sVCI group, suggesting that the alteration in rsFC might estimate the severity of cognitive impairment and that alteration in the function of the thalamus plays a role in the pathology of sVCI.

The DMN is a collection of brain regions which are typically deactivated in goal-directed tasks and activated during rest periods [58]. Providing insight into function, the default network is active when individuals are engaged in internally focused tasks including autobiographical memory retrieval, envisioning the future, and conceiving the perspectives of others [59]. As one of the part of the DMN, lateral temporal gyrus provides information from prior experiences in the form of memories and associations that are the building blocks of mental simulation[59].Other areas associated with cognitive control were also detected in the present research. The precentral gyrus and prefrontal cortex have been implicated in personality expression, decision making, planning complex cognitive behavior, planning and executing movements, and moderating social behavior, and thus changes in these regions would not be unexpected [60]. The putamen receives inputs from the association and sensorimotor areas of the cortex while the globus pallidus projects to the dorsomedial nuclei of the thalamus [61]. Sefcsik et al. [62] suggested that the frontal/subcortical circuit between the putamen and frontal motor areas plays a role in higher cognitive processing, such as executive functions, and first-order sequence learning.

## Limitations of the Study

There were some limitations in this study. First, this study used a small sample size. Second, we used clinical and not autopsy data for patient classification. AD and vascular diseases are common in old age; therefore, we unavoidably involved AD-related disease. Third, all the patients, not only VCI patients but also controls, had lacunar infarcts and white-matter hyperintensities, and because the lesions were very small (<1.5 cm) and distributed randomly throughout the brain, it was difficult to remove all of them; therefore, we processed the data as usual and did not control or consider the total number of lacunes. Fourth, although Li et al. [11] suggested that functional abnormalities can be only partly explained by morphological changes in gray-matter volume in VCI patients, the atrophy of gray-matter volume in VCI brains might have a potential impact on our results. We carefully used our own template in data preprocessing to control the effect of atrophy. Fifth, this was a cross-sectional study; therefore, it could not determine whether abnormal brain activation is a state marker or trait marker of sVCI in resting-state fMRI, and we did not follow the patients to study their functional brain changes. Finally, the correlation results were not consistent when we adopted multiple comparisons (Bonferroni correction), which means that this should only be considered an exploratory analysis. To increase the statistical power, the findings should be repeated with a larger sample of subjects.

## Conclusions

We found altered connectedness of the rsFC with PCC and VMHC in the VCI patients. This change was most likely caused by subcortical white-matter lesions that destroyed direct and indirect fiber tract connectivity across the cerebral white matter and influenced the cortical rsFC and hypoperfusion that resulted from subcortical cerebrovascular disease. rsFC correlations between the left thalamus and PCC might indicate the severity of sVCI, which might help us to understand the pathogenesis of sVCI.
